# Low salivary testosterone levels in patients with breast cancer

**DOI:** 10.1186/1471-2407-10-547

**Published:** 2010-10-11

**Authors:** Constantine Dimitrakakis, David Zava, Spyros Marinopoulos, Alexandra Tsigginou, Aris Antsaklis, Rebecca Glaser

**Affiliations:** 1 DEB, NICHD, NIH, CRC, Room 1-3330, 10 Center Drive, MSC-1103 Bethesda, Maryland, USA; 2 Athens University Medical School, 80 Vas. Sophias Av., 115 28 Athens, Greece; 3 ZRT Laboratory, Beaverton, Oregon, USA; 4 Wright State University Boonshoft School of Medicine, Dayton Ohio, USA

## Abstract

**Background:**

Correlation between circulating sex steroid levels and breast cancer has been controversial, with measurement of free, or bioavailable hormone rarely available. Salivary hormone levels represent the bioavailable fraction. To further elucidate the role of endogenous hormones in breast cancer, we aimed to assess correlation between salivary sex steroid levels and breast cancer prevalence.

**Methods:**

Salivary hormone levels of testosterone (T), Estradiol (E2), Progesterone (P), Estriol (E3), Estrone (E1), DHEAS and Cortisol (C) were measured by Enzyme Immunoassay (EIA) in 357 women with histologically verified breast cancer and 184 age-matched control women.

**Results:**

Salivary T and DHEAS levels were significantly lower in breast cancer cases vs. controls (27.2+13.9 vs. 32.2+17.5 pg/ml, p < 0.001 for T and 5.3+4.3 vs. 6.4+4.5 ng/ml, p = 0.007 for DHEAS). E2 and E1 levels were elevated and E3 levels were lowered in cases vs. controls.

**Conclusions:**

Salivary T levels, representing the bioavailable hormone, are significantly lower in women with breast cancer compared to age-matched control women. These findings support the protective role of biovailable testosterone in counteracting the proliferative effects of estrogens on mammary tissue.

## Background

The risk of developing breast cancer is related to events of reproductive life and lifestyle factors that modify levels of endogenous sex hormones. Soon after the discovery of sex hormones, it was suggested that breast cancer risk was related to endogenous estrogen levels. Recent large prospective cohort studies on postmenopausal women make it clear that high levels of estrogens increase mammary gland proliferation and are associated with increased breast cancer risk [1].

Interestingly, several observations suggest that androgens may counteract the proliferative effects of estrogen and progestogen in the mammary gland. In cell cultures and animal experiments, androgens have been shown to exert anti-proliferative effects [2]. It has also been demonstrated that a negative association exists between breast cell proliferation and levels of free testosterone in both pre- and postmenopausal women [3,4]. However, the relationship between endogenous androgen levels (testosterone, androstenedione, dehydroepiandrosterone (DHEA)) and breast cancer risk is still unclear with both epidemiologic and experimental data providing conflicting results [5,6]. Many of these studies use inaccurate methods to measure the low levels of bioactive forms of androgens in women.

Our aim was to examine whether salivary testosterone levels were lower in breast cancer patients compared to the general population. Hypotheses of whether elevated testosterone levels are associated with elevated estrogen levels were also investigated. To address this issue a case-control study was designed. Immunoassay has been used for the measurement of hormones in saliva, given the sensitivity, reliability, and serum saliva correlations for these assays [7,8].

## Methods

Salivary hormone levels were collected in 357 newly diagnosed breast cancer patients between 2001 and 2004. Breast cancer diagnosis was categorized according to histological reports to: IDC (infiltrating ductal carcinoma), DCIS (ductal carcinoma in situ), ILC (infiltrating lobular carcinoma) and LCIS (lobular carcinoma in situ, a risk factor for invasive breast cancer). All patients were in a single surgical practice of one of us (RG).

Salivary hormone levels were also collected in a group of 184 controls. These were women who presented with benign breast disease (benign breast lump, fibrocystic tissue or breast pain) in the same practice (RG), during the same time period. Patients with atypical ductal hyperplasia were excluded from the study.

The two groups (patients and controls) were matched for breast cancer risk factors such as age, menopause status, family history of breast cancer, use of hormone therapy, age at menarche and age at first birth (Table 1). Breast cancer patients had an average age of 54.8 years (ranging from 30 up to 86 years) while in the control group the average age was 53.5 years (31 to 81 years). Two hundred and thirty eight patients were menopausal (66.7%), while in the control group 115 women were menopausal (62.5%). A chi-square test indicated that there were no statistically significant differences between the two groups according to age and to menopausal status (p-value 0.115 and 0.342, respectively). The two groups were also matched for non-malignancy associated surgical menopause: 31 out of 184 (16.84%) women in the control group and 58 out of 357 (16.24%) had a surgical hysterectomy with bilateral oophorectomy. About half of the patients (47.8%) had a family member suffering from breast or ovarian cancer and this rate was comparable to the control group (51.7%, chi-square p-value 0.535). Some women in our study received hormonal treatment, either estrogen or progesterone or both, but there was no evidence that these rates differ amongst the two groups (p-value 0.066). Few patients were supplementing with estradiol, the majority had been on conjugated equine estrogens. The control group had an average age at menarche 12.7 years and the case group 12.6 years, while age at first pregnancy was 24.3 years for the control group and 23.9 years for breast cancer patients. Graphics and comparisons are illustrated in Table 1.

**Table 1:** Demographics of control vs. breast cancer patient groups.

		Controls (N = 184)	Cases (N = 357)	p-value
**Age**		53.5 (+9.7)	54.8 (+12)	0.181

**Menopausal**	pre	69 (37.5%)	119 (33.3%)	

**status**	post	115 (62.5%)	238 (66.7%)	0.342

**Family**	negative	72 (48.3%)	175 (52.2%)	

**history**	positive	77 (51.7%)	160 (47.8%)	0.535

**HRT**	no	96 (52.2%)	217 (60.8%)	

	yes	88 (47.8%)	140 (39.2%)	0.066

**Age at menarche**		12.7 (+1.3)	12.6 (+1.5)	0.319

**Age at 1^st ^pregnancy**		24.3 (+4.6)	23.9 (+5.1)	0.503

Age is in years. Standard deviation and percentages are in parenthesis. P-values were computed using chi-square tests and students t-test.

Salivary testosterone (T), Estradiol (E2), Progesterone (P), Estriol (E3), Estrone (E1), dehydroepiandrosterone sulfate (DHEAS) and cortisol (C) levels were measured in all women and compared between the two groups (Table 2). All salivary specimens were evaluated at the same laboratory (ZRT).

**Table 2:** Mean levels of hormone measurements by group and according to menopausal status.

	Controls (sd)	Cases (sd)	p-value
**T**			
**pre**	34.3 (16.1)	31.4 (15.3)	0.234
**post**	31.0 (17.6)	25.1(12.6)	0.002
**overall**	32.2 (17.5)	27.2 (13.9)	< 0.001
**E1**			
**pre**	3.8 (2.9)	5.0 (2.9)	0.037
**post**	3.6 (2.1)	4.8 (4.8)	0.047
**overall**	3.7 (2.4)	4.9 (4.2)	0.006
**E2**			
**pre**	2.1 (2.1)	2.4 (2.0)	0.295
**post**	1.2 (0.8)	1.7 (1.3)	< 0.001
**overall**	1.65 (1.3)	2.0 (1.6)	0.005
**E3**			
**pre**	5.2 (7.3)	2.7 (1.5)	0.001
**post**	3.7 (2.6)	3.6 (3.8)	0.734
**overall**	4.3 (4.9)	3.3 (3.3)	0.011
**DHEAS**			
**pre**	7.3 (4.8)	6.8 (4.9)	0.434
**post**	5.7 (4.2)	4.5 (3.7)	0.007
**overall**	6.4 (4.5)	5.3 (4.3)	0.007
**T/E1**			
**pre**	28.26 (46.7)	8.23 (7.6)	< 0.001
**post**	22.97 (79.5)	8.74 (12.9)	0.021
**overall**	24.75 (68.5)	8.57 (11.4)	0.014
**T/E2**			
**pre**	19.34 (11.6)	19.90 (23.43)	0.667
**post**	34.09 (25.5)	25.86 (52.3)	0.063
**overall**	27.45 (21.7)	23.86 (44.6)	0.212

T/E1 and T/E2, average ratios of testosterone over estrone and estradiol, respectively. Sd, standard deviation. P-values were computed using students' t-test. Testosterone (**T**) pg/ml, Estrone (**E1**) pg/ml, Estradiol (**E2**) pg/ml, Estriol (**E3**) pg/ml, DHEAS ng/ml

An ethical approval was obtained for this study from Athens University Medical School and an informed consent was obtained from the subjects. Women were fully informed on the details of the study and agreed to participate and have their data published.

### Saliva Collection

Because hormone levels vary throughout the day, leading to inaccuracies in many studies, saliva (minimum 5 ml) was collected in polypropylene tubes in the morning upon rising and before breakfast (7-9 a.m.). Food and beverages (except water) were avoided 2 h prior to saliva collection. Women on hormone replacement therapy (HRT) had discontinued therapy at least one month prior to salivary hormone collection. Saliva samples were shipped within 24 h for laboratory analysis.

### Saliva Processing

Saliva was processed by adding 50 μl of 0.14 mg/ml dithiothreitol (DTT) per ml of saliva to break up mucins that interfere with saliva extraction. Steroids were then extracted from 1.5 ml of saliva by C-18 column chromatography. Samples were gently pulled through the columns by vacuum. Control and calibrator samples were prepared from Biorad Lyphocheck diluted 1/100 in phosphate-buffered saline (PBS) buffer containing DTT. The C-18 columns were washed twice with PBS buffer, vacuum dried, and the steroids eluted with 100% ethanol. The eluted solvent containing the steroids was dried under nitrogen and then reconstituted 2× in PBS buffer containing 0.1% T904 detergent and 0.05% Proclin antimicrobial (assay buffer).

### Steroid Testing

Steroids in the extracted/reconstituted saliva were quantified by enzyme immunoassay (EIA) with commercial kits from DRG, Germany. Standards were prepared in assay buffer from a concentrated stock of each hormone with serial dilution. Inter- and intra-assay coefficients of variation for low and high controls for all steroids tested were 10% or less. Ranges were based on gender, age, menstrual status (e.g. follicular vs. luteal phase of the menstrual cycle) from greater than 1000 women in each category.

### Statistics

Univariate statistical analysis was performed to address the questions stated before.

Normality of the data was checked using graphical investigation (histograms and q-q plots) as well as the Kolmogorov Smirnov test (results not shown). Student's t-test was used to compare the mean hormone levels while chi-square tests were performed when comparing frequencies. A simple linear regression was used to illustrate graphically the correlation between testosterone and E1, E2 levels. Also, multivariate analysis was performed to assess the dependency of testosterone levels on a set of independent variables. All statistical analysis were performed in an alpha = 5% level.

## Results

Table 2 presents steroid hormone measurements for the two groups. T and DHEAS levels were significantly lower (15.5% and 17.2%, p-value <0.001 and 0.007, respectively) in breast cancer patients vs. the control group. E3 levels were also 23.2% lower in the breast cancer group (P = 0.01). On the contrary, levels of E2 and E1 were higher in breast cancer patients (17.5% and 24.5%, p-value 0.005 and 0.006, respectively). No statistically significant differences were found for the levels of progesterone or cortisol (data not shown). Comparing the number of breast cancer patients versus controls with levels of testosterone below normal (< 20 pg/ml), we have 20.1% of women in the control group and 29.6% of patients, with levels of testosterone lower than normal (p-value = 0.021).

Menopausal women had, on average, reduced T and DHEAS levels when compared with premenopausal in both groups (data not shown). Comparison of salivary hormone levels according to menopausal status (Table 2) showed that T and DHEA-S levels were markedly lower in the post-menopausal cancer group vs. post-menopausal control. (19% and 21% reduction, p-values 0.002 and 0.007 for T and DHEA-S respectively). In pre-menopause, although levels are lower in patients, it does not reach statistical significance. On the contrary, E2 levels were higher in the post-menopausal breast cancer group vs. post-menopausal controls (29.4% increase, p-value <0.001), while E1 levels were higher both in pre- and post-menopausal breast cancer patients.

Ratios of testosterone over E1 and E2 were also computed overall as well as in pre and post menopausal women (Table 2). Differences of T/E1 are significant when comparing the two groups overall (p-value 0.014) as well as the different strata (p < 0.001 and p = 0.021 for pre and post-menopausal women, respectively). When comparing the ratio of testosterone over E2 no significant differences could be found.

Mean levels of hormones were compared in breast cancer cases according to the histology type of the excised tumor: infiltrating ductal or lobular carcinoma (IDC/ILC)] or ductal carcinoma in situ (DCIS). Results are shown in Table 3. No statistically significant differences were found.

**Table 3:** Average levels of hormones according to histology type.

	Cases (sd)	p-value
**Testosterone**		

**IDC/ILC**	27.8 (14.4)	

**DCIS**	24.8 (11.3)	0.146

**E1**		

**IDC/ILC**	5.21 (4.5)	

**DCIS**	4.15 (3.3)	0.162

**E2**		

**IDC/ILC**	2.09 (1.5)	

**DCIS**	1.70 (1.2)	0.132

**E3**		

**IDC/ILC**	3.55 (3.5)	

**DCIS**	3.33 (2.3)	0.976

**DHEAS**		

**IDC/ILC**	5.38 (4.3)	

**DCIS**	5.03 (4.4)	0.585

IDC, infiltrating ductal carcinoma, ILC, infiltrating lobular carcinoma, DCIS, ductal carcinoma in situ. IDC/ILC, 298 cases, DCIS, 53 cases, 6 cases were missing. Sd, standard deviation.

Testosterone pg/ml, Estrone (**E1**) pg/ml, Estradiol (**E2**) pg/ml, Estriol (**E3**) pg/ml, DHEAS ng/ml

**Figure 1:**
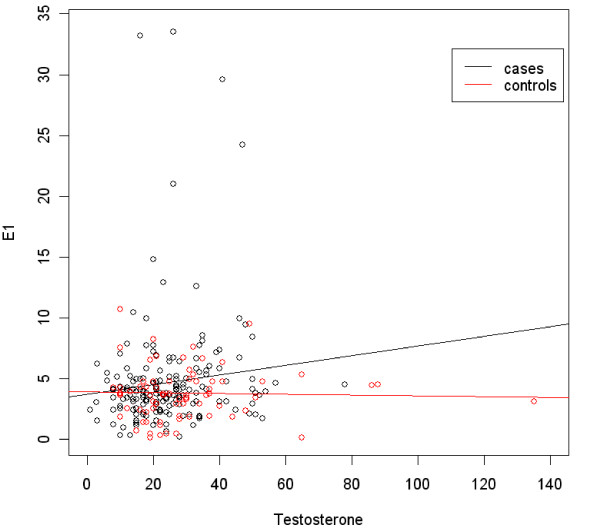
**Regression of testosterone on E1. Cases are plotted as black circles while controls as red**. Regression line equation for controls: E1 = 4.256+0.012* Testosterone, R^2 ^= 0.003 p-values <0.001, 0.532 for constant and beta1, respectively. Regression line equation for cases: E1 = 3.662+0.046*Testosterone, R^2 ^= 0.025 p-values <0.001, 0.017 for constant and beta1, respectively. E1, pg/ml, Testosterone, pg/ml.

**Figure 2:**
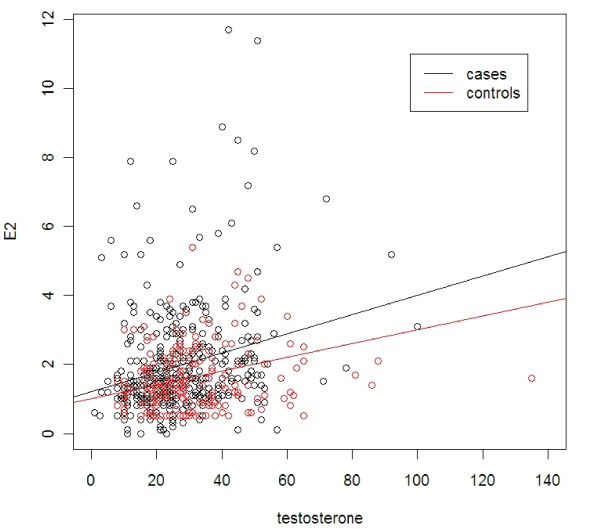
**Lines represent regression of testosterone on E2. Cases are plotted as black circles while controls as red**. Regression line equation for controls: E2 = 1.002+0.02* Testosterone, R^2 ^= 0.181 p-values 0.235, <0.001 for constant and beta1, respectively. Regression line equation for cases: E2 = 1.212+0.028*Testosterone, R^2 ^= 0.061 p-values <0.001, <0.001 for constant and beta1, respectively. E2, pg/ml, Testosterone, pg/ml.

To assess whether breast cancer patients with higher testosterone levels have corresponding higher E1 levels, a scatter plot was created. In Figure 1, measurements taken from both cancer and control groups are presented. It shows that there is a positive correlation between testosterone and E1 levels in breast cancer patients. This means that as testosterone levels increase in breast cancer patients, E1 levels tend to increase as well. The opposite holds for controls, in which the regression line has a decreasing slope, showing that as testosterone levels increase, E1 levels tend to decrease. Spearman's correlation coefficient was 0.159 (p-value 0.012) for cases and -0.030 (p-value 0.749) for controls. Figure 2 represents the relationship between Testosterone and E2 levels. In both cases and controls there is a positive correlation as indicated from the regression lines (Spearman's correlation coefficient 0.425 in control group and 0.249 in cases group, p-values <0.001 in both groups). There is also a positive correlation between E1 and E2 levels (data not shown).

A multivariate linear model was used to assess the dependency of Testosterone levels on a set of independent variables (Table 4). It was found that testosterone levels are significantly related to age (p-value 0.017), to the group that each subject belongs to (cases or controls, p-value <0.001), to E2 levels (p-value <0.001) and to progesterone levels (p-value 0.03). Looking at the values of coefficients it can be seen that testosterone levels increase by 2.78 as E2 increases, while similar positive association can be seen with progesterone. On the other hand, as age increases the levels of testosterone are dropping by 0.14 on average.

**Table 4:** Multivariate analysis showing the dependency of testosterone on a set of independent variables.

Age	-0.135	0.017
Group	-5.580	< 0.001

E2	2.381	< 0.001

progesterone	0.001	0.03

## Discussion

This study has shown for the first time that endogenous bioavailable testosterone levels are lower in women with breast cancer vs. relevant control women. Although saliva has not yet become a mainstream sample source for hormone analysis, it is a valuable research tool offering a non-invasive and stress-free alternative to plasma and serum. It has proven to be reliable and in some cases superior to other body fluids demonstrating a very close correlation with free testosterone levels in serum [9]. Saliva has several advantages over blood as a sampling medium: it can be easily collected by subjects themselves at repeated intervals; it requires no special collection or storage equipment; and the steroid concentrations measured exclude the fraction tightly bound to serum proteins and thus unavailable for biological action [8,10]. Testosterone serum levels have limited reliability in the low ranges found in normal women [11] and they vary widely based on genetic, metabolic and endocrine influences [12]. It is now accepted that measurements of free or bioavailable testosterone predicts androgenic effects more accurately than total testosterone levels. For the accuracy of our study, all samples were collected at the same time of the day. Early morning collections ensure measurement of the higher diurnal testosterone levels in all individuals.

Our findings that estrogens are related to individual breast cancer risk both premenopausally and postmenopausally are generally consistent with former studies [13-16]. In line with other authors, we report higher concentrations of estrogens being associated with increased breast cancer risk both in pre- and post-menopausal women [16,17]. Estrone and Estradiol mean levels were elevated in both pre and post menopausal patients; however E2 does not reach statistical significance in the pre-menopausal group. The latter was not unexpected given that we assessed only free estradiol levels in saliva. Free estradiol is the most potent circulating estrogen, and it is usually presenting only minimal variation [18]. Because of its highly activating potential, estradiol levels tend to be preserved almost invariably in each woman by highly sophisticated mechanisms like FSH feedback and SHBG levels. Significant differences of activated E2 levels could induce acute symptoms mimicking menarche or menopause and breast and endometrial cancers that are considered result of prolonged estrogenic action. Estriol seems to be breast cancer protective since it was found elevated in pre-menopausal controls vs. breast cancer patients. It has been reasoned that the principal pregnancy estrogen estriol and other pregnancy hormones are not associated with a risk of breast cancer [19] and they may also reduce breast cancer risk later in life [20].

There are conflicting data on the association between androgen levels and breast cancer risk [21]. Some retrospective, epidemiologic studies using inaccurate methodologies to measure testosterone have shown an increased incidence of breast cancer associated with elevated testosterone levels. Also, androgens can stimulate the breast indirectly by providing the major substrate for the synthesis of estrogens in peripheral or mammary adipose tissue. However, other studies have shown a decreased risk or no difference [3,4,17]. Using an accurate methodology, we report lower testosterone and DHEAS levels in breast cancer patients vs. controls indicating a lower androgen production in breast cancer patients than in healthy individuals. In addition, when taking the level of 20 ng/dl as the lower normal value of testosterone, there were more breast cancer patients than controls with levels below normal values.

Evidence suggests that the fall in androgen production begins as early as the third decade in women, with a gradual decline thereafter [6]. We found that T levels are significantly related to age and as age increases, the levels of T are dropping by 0.14 on average. As expected, T and DHEAS levels in our study are influenced by the menopausal status with menopausal women having, on average, reduced levels compared with premenopausal women, an observation that is in accordance with published literature [22]. Since the mechanism of hormone production changes dramatically after menopause, the two groups were stratified according to menopausal status. According to this stratification, postmenopausal breast cancer patients present with lower T and DHEAS levels when compared to controls. The same happens with premenopausal women; however, this difference was not statistically significant. Thus, the overall lower androgen levels in patients with breast cancer could be attributed to the subgroup of post-menopausal women, suggesting that breast cancer risk augments post-menopausally as testosterone levels decline.

One may argue that there are some breast cancer patients in our study with high androgen levels and some healthy individuals with low levels. We believe that higher testosterone levels are generally associated with higher estrogen levels since androgens are the substrate for conversion to estrogens. This could explain the positive association in some studies between breast cancer and testosterone where, this association was no longer significant after adjusting for estrogens [23,24]. In our study, breast cancer patients with high testosterone levels have also higher estrogen levels (Figure 1, 2). There is a positive correlation between T and E1 or E2 levels in breast cancer patients. In other words, as T levels increase in patients, estrogen levels increase as well. On the contrary, higher T levels in control patients are associated with lower E1 levels. In addition, in a multivariate analysis, T levels are significantly related not only to age but to E2 levels as well. The above observations could mean that higher testosterone concentration proved insufficient to inhibit tumorinogenesis in presence of high estrogen levels. Our findings are not in contrast with the hypothesis of Liao et al. who proposed that concomitant elevation in both androgens and estrogens may confer a greater risk for mammary gland tumorigenesis than the elevation of each hormone alone [25].

It is has also been suggested that the balance between the stimulatory effect of estrogens and the inhibitory effect of androgens is the critical factor that regulates mammary cell proliferation both in normal and in cancer tissues [22]. In support of these observations, the T/E1 ratio in our study was lower in breast cancer cases when compared to controls overall, pre and post-menopausally. The exact concentrations of estrogens and androgens in the breast and their intracrinological regulation not completely clear, nor is the fragment of the circulating androgens that may reflect the mammary tissue effective 'hormone load' [4,22,26].

Unlike other studies [27,28], no correlation was found between tumor histology and hormone levels. Testosterone and estrogen levels did not differ between in-situ and invasive carcinoma. It could mean that hormone imbalance is crucial for carcinogenesis and not for tumor progression.

A major weakness of the present study is that body mass index (BMI) was not reported. However, the relationship of adiposity with breast cancer especially in postmenopausal women could be partially explained by the increases in endogenous estrogens [29], and not by a decrease in biovailable androgens. The risk decreases after adjustment to estrogens but not to androgens [30].

Our findings are in agreement with clinical and experimental observations suggesting that androgens counteract the proliferative effects of estrogens on the mammary gland and are considered to be protective against estrogenic carcinogenesis [3,4,31,32]. In the present study, bioavailable testosterone levels in breast cancer patients were found to be statistically significantly lowered compared to non-cancer controls, supporting the hypothesis of an 'androgen-protection deficiency' occurring in breast cancer patients.

## Conclusions

Salivary testosterone levels are significantly lower in breast cancer patients compared to controls. These differences are more profound in postmenopausal women. Salivary E1 and E2 levels were elevated in cases leading to a lower T/E1 ratio in breast cancer patients comparing to healthy individuals.

Our results support a correlation between endogenous androgen and estrogen levels and breast cancer risk. Breast cancer patients appear to have a relative imbalance of sex steroid hormones in favor of estrogens. Higher biovailable testosterone may counteract the proliferative effects of estrogens on mammary tissue and may exert a protective role to the breast, inhibiting cancer development and/or tumor growth.

## Abbreviations

DHEAS: Dehydroepiandrosterone Sulfate; FSH: Follicular Stimulating Hormone; SHBG:Sex Hormone Binding Globulin; T:Testosterone; E1:Estrone; E2: Estradiol; E3: Estriol; P:Progesterone; C:Cortisol; EIA:Enzyme ImunoAssay; DTT:Dithiothreitol; PBS:Phosphate-buffered saline; IDC:Infiltrating Ductal Carcinoma; ILC:Infiltrating; Lobular Carcinoma; DCIS:Ductal Carcinoma in situ; LCIS:Lobular Carcinoma in situ; HRT:Hormone Replacement Therapy; BMI:Body Mass Index; pg/ml: picograms per mililiter; ng/ml: nanograms per mililiter.

## Competing interests

The authors declare that they have no competing interests.

## Authors' contributions

**CD **participated in supervising, analyzing data, writing and literature search**, DZ **made the lab analysis, **SM **made the statistics**, AT **participated in literature search and writing**, AA **participated in editing the ms**, RG **had the conception of the study and recruited participants. All authors read and approved the final manuscript.

## Pre-publication history

The pre-publication history for this paper can be accessed here:

http://www.biomedcentral.com/1471-2407/10/547/prepub

## References

[B1] Henderson BE Feigelson HS Hormonal carcinogenesis Carcinogenesis 2000 21 3 427 433 10.1093/carcin/21.3.427 10688862

[B2] Ando S De Amicis F Rago V Carpino A Maggiolini M Panno ML Lanzino M Breast cancer: from estrogen to androgen receptor Mol Cell Endocrinol 2002 193 1-2 121 128 10.1016/S0303-7207(02)00105-3 12161011

[B3] Hofling M Hirschberg AL Skoog L Tani E Hagerstrom T von Schoultz B Testosterone inhibits estrogen/progestogen-induced breast cell proliferation in postmenopausal women Menopause 2007 14 2 183 190 10.1097/01.gme.0000232033.92411.51 17108847

[B4] Dimitrakakis C Zhou J Wang J Belanger A LaBrie F Cheng C Powell D Bondy C A physiologic role for testosterone in limiting estrogenic stimulation of the breast Menopause 2003 10 4 292 298 10.1097/01.GME.0000055522.67459.89 12851512

[B5] Lillie EO Bernstein L Ursin G The role of androgens and polymorphisms in the androgen receptor in the epidemiology of breast cancer Breast Cancer Res 2003 5 3 164 173 10.1186/bcr593 12793900 PMC165007

[B6] Davison S Davis SR Hormone replacement therapy: current controversies Clin Endocrinol (Oxf) 2003 58 3 249 261 10.1046/j.1365-2265.2003.01774.x 12608928

[B7] Krause W Mueller U Mazur A Measurement of steroid levels in saliva in a population-based survey of lifestyle, medical conditions, marriage, sex life and hormone status in aging men: a feasibility study Aging Male 2002 5 4 203 215 12630067

[B8] Glaser RL Zava DT Wurtzbacher D Pilot study: absorption and efficacy of multiple hormones delivered in a single cream applied to the mucous membranes of the labia and vagina Gynecol Obstet Invest 2008 66 2 111 118 10.1159/000128599 18446040

[B9] Groschl M Current status of salivary hormone analysis Clin Chem 2008 54 11 1759 1769 10.1373/clinchem.2008.108910 18757583

[B10] Cook CJ Rapid noninvasive measurement of hormones in transdermal exudate and saliva Physiol Behav 2002 75 1-2 169 181 10.1016/S0031-9384(01)00658-8 11890965

[B11] Lobo RA Androgens in postmenopausal women: production, possible role, and replacement options Obstet Gynecol Surv 2001 56 6 361 376 10.1097/00006254-200106000-00022 11466487

[B12] Tchernof A Despres JP Sex steroid hormones, sex hormone-binding globulin, and obesity in men and women Horm Metab Res 2000 32 11-12 526 536 10.1055/s-2007-978681 11246820

[B13] Clemons M Goss P Estrogen and the risk of breast cancer N Engl J Med 2001 344 4 276 285 10.1056/NEJM200101253440407 11172156

[B14] Rod NH Hansen AM Nielsen J Schnohr P Gronbaek M Low-risk factor profile, estrogen levels, and breast cancer risk among postmenopausal women Int J Cancer 2009 124 8 1935 1940 10.1002/ijc.24136 19123466

[B15] Cummings SR Tice JA Bauer S Browner WS Cuzick J Ziv E Vogel V Shepherd J Vachon C Smith-Bindman R Prevention of breast cancer in postmenopausal women: approaches to estimating and reducing risk J Natl Cancer Inst 2009 101 6 384 398 10.1093/jnci/djp018 19276457 PMC2720698

[B16] Eliassen AH Missmer SA Tworoger SS Spiegelman D Barbieri RL Dowsett M Hankinson SE Endogenous steroid hormone concentrations and risk of breast cancer among premenopausal women J Natl Cancer Inst 2006 98 19 1406 1415 10.1093/jnci/djj376 17018787

[B17] Adly L Hill D Sherman ME Sturgeon SR Fears T Mies C Ziegler RG Hoover RN Schairer C Serum concentrations of estrogens, sex hormone-binding globulin, and androgens and risk of breast cancer in postmenopausal women Int J Cancer 2006 119 10 2402 2407 10.1002/ijc.22203 16894564

[B18] Laven JS Fauser BC What role of estrogens in ovarian stimulation Maturitas 2006 54 4 356 362 10.1016/j.maturitas.2006.04.022 16782289

[B19] Lyytinen H Pukkala E Ylikorkala O Breast cancer risk in postmenopausal women using estrogen-only therapy Obstet Gynecol 2006 108 6 1354 1360 17138766 10.1097/01.AOG.0000241091.86268.6e

[B20] Jacobson HI Lemanski N Narendran A Agarwal A Bennett JA Andersen TT Hormones of pregnancy, alpha-feto protein, and reduction of breast cancer risk Adv Exp Med Biol 2008 617 477 484 full_text 18497072 10.1007/978-0-387-69080-3_47

[B21] Dimitrakakis C Bondy C Androgens and the breast Breast Cancer Res 2009 11 5 212 10.1186/bcr2413 19889198 PMC2790857

[B22] Labrie F Luu-The V Labrie C Belanger A Simard J Lin SX Pelletier G Endocrine and intracrine sources of androgens in women: inhibition of breast cancer and other roles of androgens and their precursor dehydroepiandrosterone Endocr Rev 2003 24 2 152 182 10.1210/er.2001-0031 12700178

[B23] Zeleniuch-Jacquotte A Shore RE Koenig KL Akhmedkhanov A Afanasyeva Y Kato I Kim MY Rinaldi S Kaaks R Toniolo P Postmenopausal levels of oestrogen, androgen, and SHBG and breast cancer: long-term results of a prospective study Br J Cancer 2004 90 1 153 159 10.1038/sj.bjc.6601517 14710223 PMC2395327

[B24] Somboonporn W Davis SR Testosterone effects on the breast: implications for testosterone therapy for women Endocr Rev 2004 25 3 374 388 10.1210/er.2003-0016 15180949

[B25] Liao DJ Dickson RB Roles of androgens in the development, growth, and carcinogenesis of the mammary gland J Steroid Biochem Mol Biol 2002 80 2 175 189 10.1016/S0960-0760(01)00185-6 11897502

[B26] Sasano H Suzuki T Miki Y Moriya T Intracrinology of estrogens and androgens in breast carcinoma J Steroid Biochem Mol Biol 2008 108 3-5 181 185 10.1016/j.jsbmb.2007.09.012 17933521

[B27] Daling JR Malone KE Doody DR Voigt LF Bernstein L Coates RJ Marchbanks PA Norman SA Weiss LK Ursin G Relation of regimens of combined hormone replacement therapy to lobular, ductal, and other histologic types of breast carcinoma Cancer 2002 95 12 2455 2464 10.1002/cncr.10984 12467057

[B28] Calle EE Feigelson HS Hildebrand JS Teras LR Thun MJ Rodriguez C Postmenopausal hormone use and breast cancer associations differ by hormone regimen and histologic subtype Cancer 2009 115 5 936 945 10.1002/cncr.24101 19156895

[B29] Rinaldi S Key TJ Peeters PH Lahmann PH Lukanova A Dossus L Biessy C Vineis P Sacerdote C Berrino F Anthropometric measures, endogenous sex steroids and breast cancer risk in postmenopausal women: a study within the EPIC cohort Int J Cancer 2006 118 11 2832 2839 10.1002/ijc.21730 16385576

[B30] Key TJ Appleby PN Reeves GK Roddam A Dorgan JF Longcope C Stanczyk FZ Stephenson HE Jr Falk RT Miller R Body mass index, serum sex hormones, and breast cancer risk in postmenopausal women J Natl Cancer Inst 2003 95 16 1218 1226 10.1093/jnci/djg022 12928347

[B31] Gelfand MM It might be wise to consider adding androgen to the estrogen or estrogen-progestin regimens in the appropriate patients Menopause 2004 11 5 505 507 10.1097/01.GME.0000135245.27220.66 15356402

[B32] Dimitrakakis C Jones RA Liu A Bondy CA Breast cancer incidence in postmenopausal women using testosterone in addition to usual hormone therapy Menopause 2004 11 5 531 535 10.1097/01.GME.0000119983.48235.D3 15356405

